# *Ex vivo* anti-inflammatory effects of probiotics for periodontal health

**DOI:** 10.1080/20002297.2018.1502027

**Published:** 2018-07-25

**Authors:** Tim Schmitter, Bernd L. Fiebich, Joerg T. Fischer, Max Gajfulin, Niklas Larsson, Thorsten Rose, Marcus R. Goetz

**Affiliations:** a Symrise AG, Holzminden, Germany; b VivaCell Biotechnology GmbH, Denzlingen, Germany; c Probi AB, Lund, Sweden

**Keywords:** Gingivitis, inflammation, bacteria, oral hygiene, dental plaque, biofilms, probiotics

## Abstract

**Background**: Probiotic bacteria with anti-inflammatory properties have the potential to be of therapeutic benefit in gingivitis.

**Objective**: To evaluate the effects of potential probiotic strains on inflammatory mediators involved in early gingivitis using an *ex vivo* inflammation model.

**Methods**: Strains were screened in viable and attenuated forms for effects on bacterial lipopolysaccharide (LPS)-stimulated release of interleukins (IL)-1β, -6 and -8, tumor necrosis factor-α, prostaglandin E_2_ and 8-isoprostane from human primary monocytes, and then, if anti-inflammatory effects were shown, on IL-1β-stimulated release of inflammatory mediators from primary gingival fibroblasts. Lead strains were evaluated for optimal dosing, batch-to-batch variation and functional consistency in toothpaste.

**Results**: Twenty-one of 73 strains showed anti-inflammatory effects in monocytes; of which, seven showed effects in both viable and attenuated forms. Seven of 14 strains showed effects in fibroblasts. Strains *Lactobacillus paracasei* LPc-G110(SYBIO-15) and *Lactobacillus plantarum* GOS42(SYBIO-41) induced statistically significant dose-dependent reductions in the release of multiple inflammatory mediators from monocytes, which were consistent across batches. Viable *L. paracasei* LPc-G110 tooth paste significantly reduced IL-6, IL-8 and prostaglandin E_2_ release from monocytes versus placebo.

**Conclusion**: Strains *L. paracasei* LPc-G110 and *L. plantarum* GOS42 have potential for use as probiotics in oral care products to reduce gingival inflammation.

## Introduction

Inflammation of the gingivae (gingivitis) occurs in response to the accumulation of dental plaque on tooth surfaces near the gingival margin. In particular, bacterial lipopolysaccharide (LPS) from Gram-negative bacteria, which increase in number and proportion in plaque as it matures, provokes a non-specific inflammatory immune response [1,2]. This response is mediated by proinflammatory cytokines (e.g. tumor necrosis factor-α [TNF-α] and interleukin [IL]-1β), chemokines (e.g. IL-8) and prostaglandins (e.g. prostaglandin E_2_ [PGE_2_]) secreted by gingival epithelial cells, fibroblasts and resident leukocytes [2–4]. These inflammatory mediators influence various cellular processes, including recruitment and chemotaxis of neutrophils, and promote increased vascular dilation and blood flow in the gingiva [5]. Persistent gingival inflammation can progressively exert selective pressure for the development of a dysbiotic and inflammophilic plaque microbiota [6]. In susceptible individuals, these changes can lead to chronic periodontitis, which is characterized by chronic inflammation and irreversible destruction of the supporting tissues of the teeth. Therefore, gingival inflammation should ideally be prevented, or reversed in its early stages.

Prevention and treatment of gingivitis are possible by the regular practice of oral hygiene measures to remove or control dental plaque, such as tooth brushing, interdental cleaning, and the use of oral care products containing antimicrobial agents. However, these approaches are indiscriminate and may remove bacteria that are potentially beneficial to the host through, for example, their ability to reduce nitrate, antagonize exogenous pathogens or oral disease-associated species, and/or modulate the immune response to maintain homeostasis [7–9].

Some commensal bacteria of the lower gut and oral cavity, such as lactobacilli and bifidobacteria, have been shown to have anti-inflammatory properties and to reduce the symptoms of chronic inflammatory bowel disease when administered as probiotics [10]. Selected species of these genera may, therefore, be of therapeutic benefit in gingivitis. However, the effects of these bacteria on the release of inflammatory mediators associated with gingival inflammation are likely strain specific, and some commercially available strains used as probiotics may have proinflammatory effects [11,12]. Indeed, a commercially available yoghurt containing *Lactobacillus bulgaricus* and *Streptococcus thermophilus*, with additional *Lactobacillus casei* DN 114 001, was shown to enhance LPS-stimulated production of proinflammatory cytokines, including TNF-α and IL-1β, in blood culture [12].

To date, relatively few studies have investigated the effects of probiotic bacteria on the release of inflammatory mediators that play a role in the initial stages of gingivitis. In a clinical study, the use of chewing gum containing *Lactobacillus reuteri* ATCC 55,730 or ATCC PTA5289 over a two-week period was associated with significant reductions in TNF-α and IL-8 in gingival crevicular fluid (GCF) from baseline in 42 subjects with moderate gingivitis [13]. However, no differences were reported in these levels for the probiotic group versus the placebo group. In an experimental gingivitis study, daily intake of lozenges containing *L. reuteri* (ATCC 55,730 and ATCC PTA5289) did not significantly affect the levels of seven inflammatory mediators in GCF compared with a placebo control in 18 healthy adults who abstained from oral hygiene for three weeks [14]. However, topical treatment of mice with *Lactobacillus brevis* CD2-containing lyopatches resulted in a significantly lower expression of TNF, IL-1β, IL-6 and IL-17A compared to placebo in experimentally induced periodontitis [15].

The research described herein is part of a larger study to develop probiotics for oral health on a rational basis. Other properties being evaluated for individual probiotic strains include antagonism to, and co-aggregation with, disease-associated oral bacteria, and effects on the composition of oral biofilms. Strains that show promising activity in these experiments are to be tested in a proof-of-principle clinical trial. The overall aim of this study was to evaluate the effects of potential probiotic strains on inflammatory mediators involved in early gingivitis using an *ex vivo* inflammation model. Specific aims were to:

- Screen a large panel of viable and attenuated strains for anti-inflammatory effects.
- For selected strains found to be anti-inflammatory in initial screening, determine their optimal doses, evaluate batch-to-batch consistency of effects, and assess functional consistency when formulated into toothpaste.

## Materials and methods

### Selection of probiotic strains

Criteria used to select strains for screening were that they had not been genetically modified, had some prior documented anti-inflammatory activity (where available), and, preferably, had been isolated from the oral cavity; strains not isolated from the oral cavity were from food sources (e.g. fermented foods). Strains included were from the bacterial genera *Bacillus, Bifidobacterium, Lactobacillus, Lactococcus* and *Streptococcus*. Screening of strains for anti-inflammatory effects was performed sequentially in primary human monocytes and then, if some effects were shown, in primary human gingival fibroblasts, as described in the following text.

### Cultivation and attenuation of probiotic strains

#### Probiotic growth

The frozen (−80°C) probiotic stocks were kept at 4°C overnight. Then, 6 ml of sterile 9% NaCl solution was added to 1.2 ml of bacteria. The samples were centrifuged (5 min, 5000 rpm), the supernatant discarded, the pellet washed with 8 ml 9% NaCl and centrifuged again for 5 min at 5000 rpm. The pellet was resuspended in 1.2 ml 9% NaCl and 1 ml of the sample added to 50 ml of 37°C warm media (MRS Bouillon, X925.2, Carl Roth KG, Karlsruhe, Germany) and incubated at 37°C. The incubation was performed in a sterile 50 ml polypropylene tube (227,261, Greiner BioOne, Munich, Germany) and probes were harvested at different time points to evaluate the growth curves. The optical density was determined using a spectrophotometer at 600 nm (ThermoScientific, Helios Epsilon): 500 µl of bacterial suspension was diluted in 1 ml MRS Bouillon in a 1.5 ml-PS-Brand cuvette for measurement, with 1.5 ml MRS Bouillon as a blank.

#### Attenuation of probiotics

At the end of the bacterial growth log phase, 25 ml of the bacterial suspension was removed, added to a fresh 50 ml tube and incubated for 5 min at 80°C in a water bath to attenuate the probiotics.

### Preparation of human cells

#### Primary monocytes

Monocytes were extracted from the whole blood of medically healthy volunteers, who provided written informed consent at the local blood bank (University Hospital of Freiburg, Germany), following a standardized protocol (gradient preparation, lymphocytes separation medium, PAN Biotech, P04-60,125, Aidenbach, Germany) using completely endotoxin-free cultivation [16,17]. Using 50 ml tubes, 25 ml Ficoll was loaded with 25 ml of blood (buffy coats). The gradient was established by centrifugation at 1800 rpm, 20°C for 40 min with slow acceleration and deceleration. Peripheral blood mononuclear cells in the interphase were carefully removed and resuspended in 50 ml prewarmed phosphate-buffered saline (PBS) (Pan Biotech, P04-36,500), followed by centrifugation for 10 min at 1600 rpm and 20°C. The supernatant was discarded and the pellet washed in 50 ml PBS and centrifuged as mentioned earlier. The pellet was then re-suspnded in 50 ml RPMI-1640 low-endotoxin medium supplemented with 10% human serum (Hexcell, Berlin, Germany, SP2080). After counting the number of cells in a particle counter (Euro Diagnostics, Krefeld, Germany), cells were seeded in 24-well plates (2.2 million cells/well) for enzyme-linked immunosorbent assay (ELISA) or 96-well plates (200,000 cells/well) for cell viability testing and incubated at 37°C with 5% CO_2_. The medium and the non-adherent cells (lymphocytes) were removed and fresh RPMI-1640 medium containing 1% human serum was added. Enriched monocytes were thus ready for use in the experiments.

#### Primary gingival fibroblasts

Primary human gingival fibroblasts (from ProVitro, No. 1,210,412, Berlin, Germany) were maintained in a specific growth medium supplemented with growth factors (ProVitro 2,010,401) and seeded in 24-well plates (80,000 cells/well) for application of probiotics.

### Application of probiotics to monocytes and gingival fibroblasts

Human monocytes or gingival fibroblasts were incubated with concentrations of 0.1%, 0.5%, 1% and 5% v/v of the probiotics (viable or attenuated) for 30 min. Bacterial LPS (10 ng/ml; LPS from *Salmonella enterica* serotype *typhimurium* SL1181, Sigma #L9516 Taufkirchen, Germany) was then added to monocytes, and IL-1β (10 U/ml; Roche 11,457,756,001, Mannheim, Germany) to gingival fibroblasts, for an additional 24 h to stimulate an inflammatory immune response. Thereafter, supernatants were harvested, centrifuged and inflammatory parameters assessed.

### Preparation of probiotic toothpastes

Probiotic toothpastes were prepared using an adapted toothpaste formulation without ingredients that could have harmed the incorporated probiotics (e.g. sodium lauryl sulfate or methyl paraben) or interacted with the test system (e.g. silica). The probiotic toothpaste formulations are shown in Table 1 and were prepared as follows: Saccharin, sodiummonofluorophosphate, trisodiumphosphate and polyethylene glycol 1500 were first mixed in a beaker for 1 min and added along with demineralized water to a solution of sorbitol. The mixture was stirred with a magnetic stirrer until all solid particles dissolved, and carefully heated if necessary. The solution was cooled to room temperature and sodium carboxymethylcellulose was added portion-wise with vigorous stirring for 5 min to avoid clumping. Lyophilized probiotic cultures were then added and the solution stirred for a further 5 min to ensure probiotics were adequately dispersed in the gel-like formulation. For the preparation of toothpaste with attenuated probiotic, the procedure was the same except that a sorbitol-probiotic mixture was prepared before adding the other components: the sorbitol was heated up to 80°C, at which point the probiotics were added slowly under vigorous stirring. The mixture was held at 80°C for 8 min (extended to 12 min for solutions with 10% probiotic) and then cooled to 40°C. Following preparation, all toothpaste formulations were stored in glass bottles with screw caps at 2–8°C.

**Table 1.** Probiotic toothpaste formulations.

Component	Probiotic content				
	Placebo	0.50%	2.00%	5.00%	10.00%
	in % [w/w]				
Sorbitol 70%	81.79	81.39	80.16	77.70	73.61
Water, dem.	8.84	8.80	8.66	8.40	7.96
Saccharin	0.26	0.26	0.25	0.24	0.23
Sodiummonofluorophosphate	1.49	1.48	1.46	1.41	1.34
Trisodiumphosphate	0.13	0.13	0.13	0.12	0.12
PEG 1500	6.50	6.46	6.37	6.17	5.85
Sodium-Carboxymethylcellulose	1.00	1.00	0.98	0.95	0.90
Probiotic	0.00	0.50	2.00	5.00	10.00

dem. = demineralized; PEG = polyethylene glycol; w = weight

Prior to testing in monocytes, the viability of probiotics in the toothpaste was assessed using the spread plate technique. Attenuated probiotic toothpastes were checked for the absence of viable probiotic cultures; to be considered successful, the viable count had to be < 10^3^ colony forming units/g.

### Application of toothpastes to monocytes

The probiotic toothpastes were weighed and the appropriate doses diluted in cell culture media. The mixture of toothpaste in media was then placed in a 3.0 µm transwell insert (Merck Chemicals, PITP01250, Schwalbach, Germany), added to the 24-well plates containing monocytes, and stirred with the medium in the well. The cells were then incubated with the toothpaste for 30 min, after which the inserts were removed and LPS was added to the cells to stimulate the immune response. After 24 h, supernatants were harvested, centrifuged, and inflammatory parameters assessed.

### Determination of cell viability

Cytotoxicity was analyzed by Alamar Blue staining (formazan). Cells were plated in 24-well (toothpaste) or 96-well plates (pure probiotic strains) and treated with the respective test items for 24 h. Then, cells were washed once with 100 µl PBS, and 100 µl of medium-Alamar Blue-Mix (90% medium, 10% Alamar Blue, DAL1025, Thermo Fisher) was then added to each well. The plate was incubated at 37°C for 2 h in a humidified 5% CO_2_ atmosphere, and the color reaction determined using a 96-well plate reader (excitation 544 nm, emission 590 nm).

### Assessment of inflammatory parameters

Concentrations of IL-1β, IL-6, IL-8, TNF-α, PGE_2_ and 8-isoprostane were determined in monocyte supernatants, and IL-6, IL-8, PGE_2_ and 8-isoprostane in fibroblast supernatants, by enzyme immunoassays (for PGE_2_ [AssayDesign] and 8-isoprostane [Cayman]) or ELISAs (all cytokines, eBioscience: 88–8086-88 Human IL-8, 88–7346-88 Human TNF-α, 88–7066-88 Human IL-6, 88–7261-88 Human IL-1β; Immunotools, 31,670,089 Human IL-8, 31,673,019 Human TNF-α and 31,670,069 Human IL-6 Immunotools) following the manufacturer’s instructions.

### Statistical analysis

Differences in the concentrations of inflammatory mediators in supernatants from probiotic-exposed human primary cells and controls (cells exposed to IL-1β/LPS only or placebo toothpaste) were assessed using matched one-way analysis of variance tests, followed by Dunnett’s test for multiple comparisons. The level of significance was set at *P *< 0.05.

## Results

### Anti-inflammatory screening experiments

**Figure 1.**
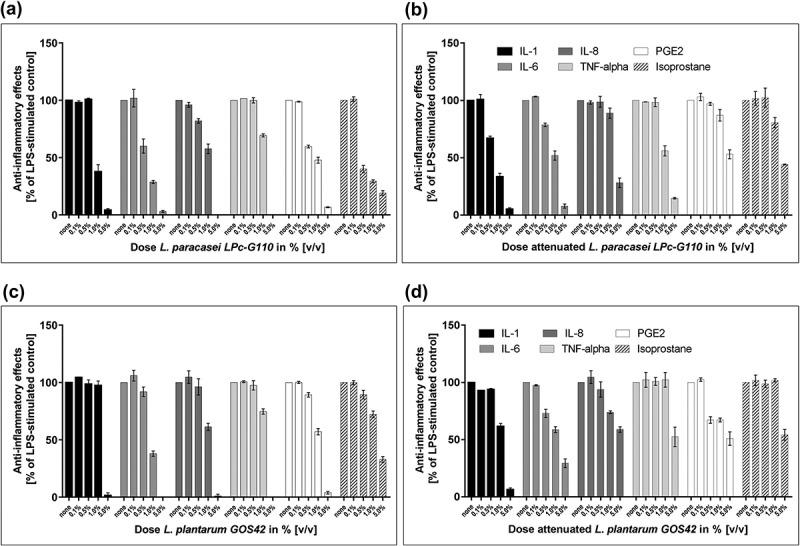
Screening of probiotic strains for effects on lipopolysaccharide (LPS)-stimulated inflammatory mediators in primary monocytes. Effects of different concentrations of *Lactobacillus paracasei* LPc-G110 in viable (a) and attenuated (b) forms. Effects of different concentrations of *Lactobacillus plantarum* GOS42 in viable (c) and attenuated (d) forms. Graphs show single experiments with biological replicates. Data are expressed as means ± standard deviation. The colony forming units per ml corresponding to each concentration of each strain are shown in Supplementary Table 1.

Seventy-three potential probiotic strains (56 *Lactobacillus* spp., 8 *Bifidobacterium* spp., 7 *Streptococcus* spp., 1 *Bacillus* sp. and 1 *Lactococcus* sp.) were screened, both in viable and attenuated forms. Twenty-one of the strains (20 *Lactobacillus* spp. and 1 *Bifidobacterium* sp.) showed anti-inflammatory effects in primary monocytes. Of these 21 strains, seven showed effects in both viable and attenuated forms. The results for strains *L. paracasei* LPc-G110(SYBIO-15) and *L. plantarum* GOS42(SYBIO-41), for which the anti-inflammatory effects were particularly pronounced, are shown in Figure 1. Viable *L. paracasei* LPc-G110 induced a reduction in LPS-stimulated release of all six inflammatory mediators at probiotic concentrations of 5% and 1% v/v (Figure 1(a)). At a concentration of 0.5%, reductions were seen for IL-6, IL-8, PGE_2_ and 8-isoprostane, while negligible effects were seen at 0.1%. These anti-inflammatory effects were mostly retained for attenuated *L. paracasei* LPc-G110, but slightly less pronounced (Figure 1(b)). An exception to this was the reduction in IL-1β, which was induced at a lower concentration of attenuated (0.5%) than viable (1.0%) *L. paracasei* LPc-G110. Viable *L. plantarum* GOS42 induced a reduction in all six mediators at a concentration of 5% and a reduction in all but one (IL-1β) at 1% (Figure 1(c)); concentrations of 0.5% or lower had little or no effect on the mediators. Attenuated *L. plantarum* GOS42 showed similar results, but with a marked reduction of IL-6 and PGE_2_ at a concentration of 0.5% (Figure 1(d)).

**Figure 2.**
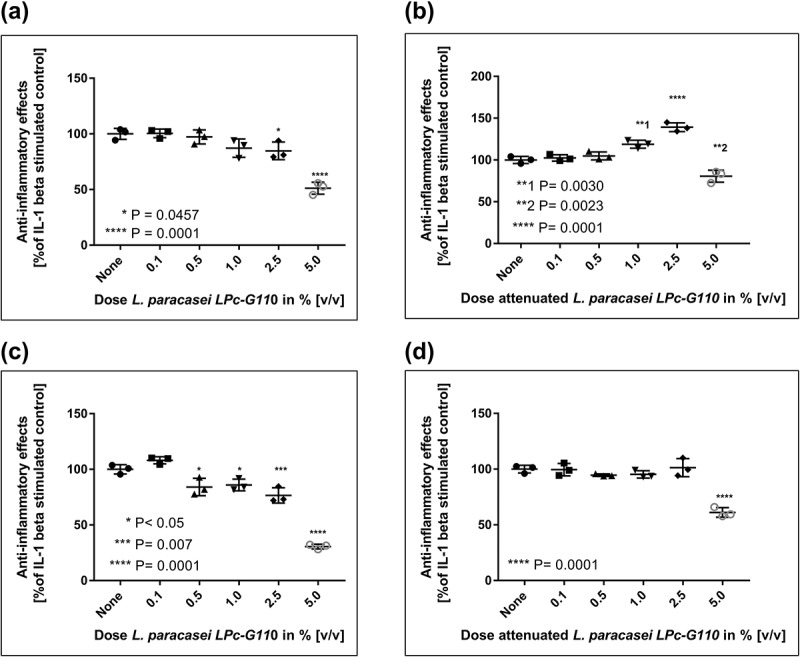
Effects of *Lactobacillus paracasei* LPc-G110 on interleukin (IL) -1β-stimulated release of IL-6 and IL-8 from gingival fibroblasts. Effects of viable (a) and attenuated (b) *L. paracasei* LPc-G110 on IL-6. Effects of viable (c) and attenuated (d) *L. paracasei* LPc-G110 on IL-8. Graphs show single experiments with biological triplicates. P-values are from matched one-way analysis of variance followed by Dunnett’s multiple comparisons test – compared to IL-1β-stimulated control. The colony forming units per ml corresponding to each concentration of *L. paracasei* LPc-G110 are shown in Supplementary Table 2.

**Figure 3.**
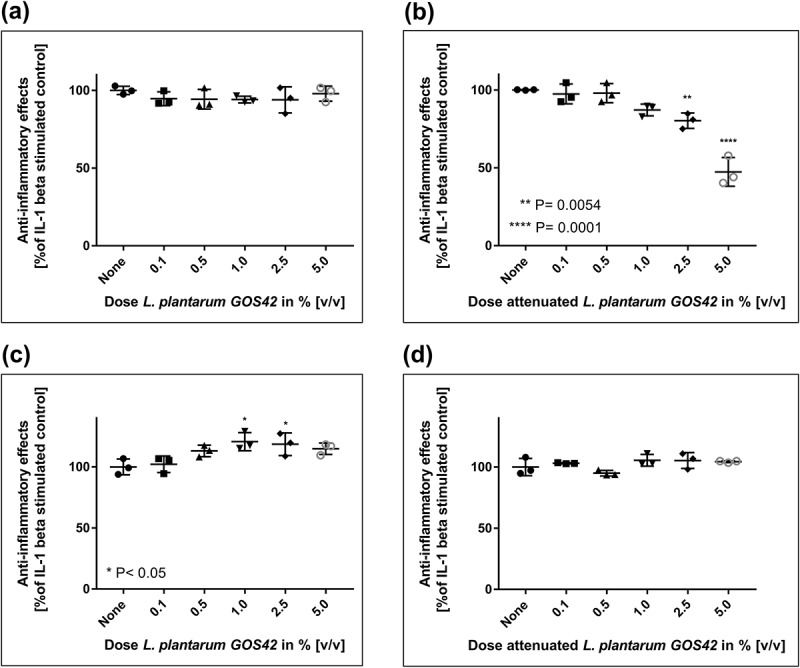
Effects of *Lactobacillus plantarum* GOS42 on interleukin (IL) -1β-stimulated release of IL-6 and IL-8 from gingival fibroblasts. Effects of viable (a) and attenuated (b) *L. plantarum* GOS42 on IL-6. Effects of viable (c) and attenuated (d) *L. plantarum* GOS42 on IL-8. Graphs show single experiments with biological triplicates. *P* values are from matched one-way analysis of variance followed by Dunnett’s multiple comparisons test – compared to IL-1β-stimulated control. The colony forming units per ml corresponding to each concentration of *L. plantarum* GOS42 are shown in Supplementary Table 2.

Fourteen of the 21 strains that showed anti-inflammatory effects in monocytes were selected for further screening in gingival fibroblasts, based on additional factors such as commercial availability, intellectual property protection, and coaggregation. Of these 14 strains, seven (all *Lactobacillus* spp.) showed anti-inflammatory effects in gingival fibroblasts. The effects of *L. paracasei* LPc-G110 and *L. plantarum* GOS42 on IL-6 and IL-8 are shown in Figures 2 and 3: Application of 5% v/v *L. paracasei* LPc-G110, both in viable and attenuated forms, significantly reduced the release of both mediators (Figure 2(a, b, c, d)), while *L. plantarum* GOS42 only induced a significant reduction in IL-6 release, in its attenuated form at 2.5% and 5% v/v (Figure 3(b)). A reduction in release of PGE_2_ and 8-isoprostane was only seen for *L. paracasei* LPc-G110 (viable or attenuated), at a concentration of 5% (results not shown).

### Follow-up experiments on anti-inflammatory strains

**Figure 4.**
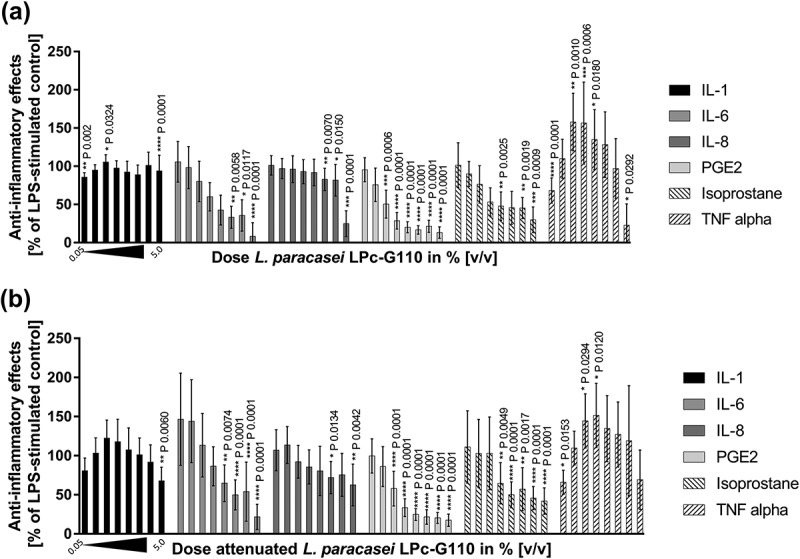
Dose-dependency of effects of *Lactobacillus paracasei* LPc-G110 on lipopolysaccharide (LPS)-stimulated release of inflammatory mediators from primary monocytes. Effects of viable (a) and attenuated (b) *L. paracasei* LPc-G110 at increasing concentrations (0.05%, 0.1%, 0.25%, 0.5%, 0.75%, 1.0%, 2.5%, 5.0% v/v); graphs show results from four independent experiments, each performed with biological triplicates and technical duplicates (*n* = 6 per experiment). All data are expressed as means ± standard deviation. P-values are from matched one-way analysis of variance followed by Dunnett’s multiple comparisons test – compared to LPS-stimulated control. The colony forming units per ml corresponding to each concentration of *L. paracasei* LPc-G110 are shown in Supplementary Table 3.

**Figure 5.**
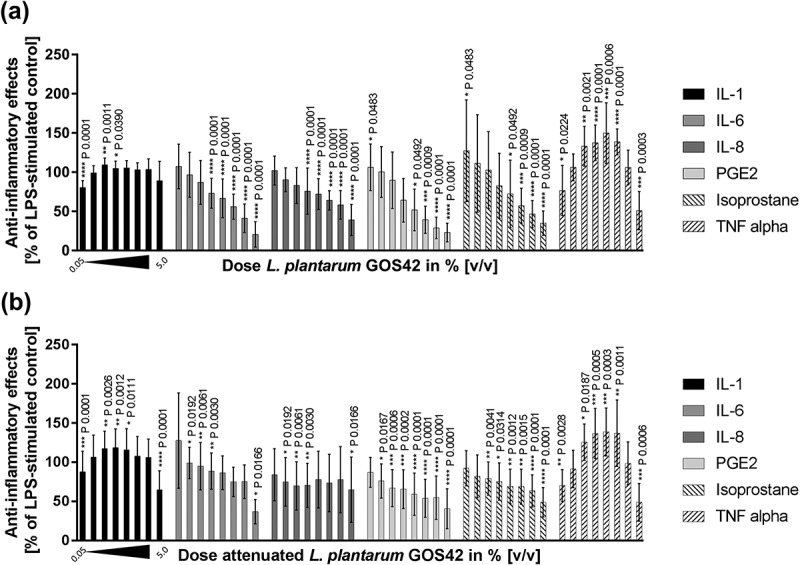
Dose-dependency of effects of *Lactobacillus plantarum* GOS42 on lipopolysaccharide (LPS)-stimulated release of inflammatory mediators from primary monocytes. Effects of viable (a) and attenuated (b) *L. plantarum* GOS42 at increasing concentrations (0.05%, 0.1%, 0.25%, 0.5%, 0.75%, 1.0%, 2.5%, 5.0% v/v); graphs show results from three independent experiments, each performed with biological triplicates and technical duplicates (*n* = 6 per experiment). All data are expressed as means ± standard deviation. *P* values are from matched one-way analysis of variance followed by Dunnett’s multiple comparisons test – compared to LPS-stimulated control. The colony forming units per ml corresponding to each concentration of *L. plantarum* GOS42 are shown in Supplementary Table 4.

**Figure 6.**
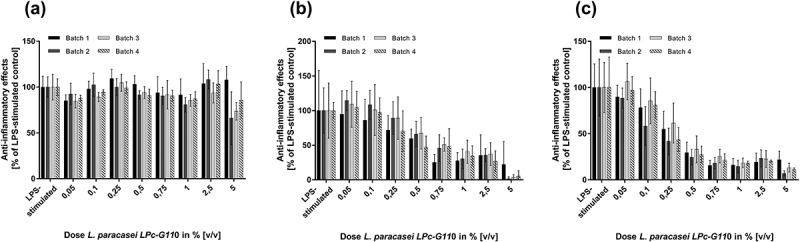
Batch-to-batch variation of effects of viable *Lactobacillus paracasei* LPc-G110 on lipopolysaccharide (LPS)-stimulated inflammatory mediators in primary monocytes. Graphs show the effects of four different batches of viable *L. paracasei* LPc-G110 at different concentrations on interleukins (IL) -1β (a) and -6 (b), and prostaglandin E_2_ (PGE_2_) (c); results are from four independent experiments, each performed with biological triplicates and technical duplicates (*n* = 6 per experiment). All data are expressed as means ± standard deviation. The colony forming units per ml corresponding to each concentration for each batch are shown in Supplementary Table 3.

**Figure 7.**
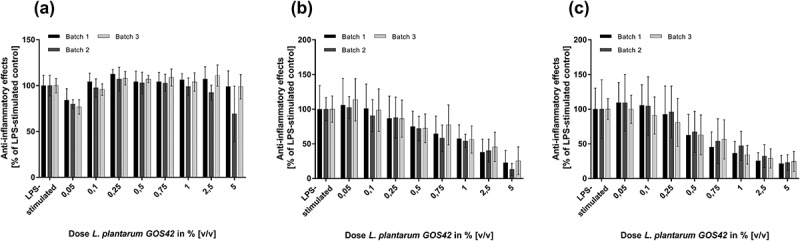
Batch-to-batch variation of effects of viable *Lactobacillus plantarum* GOS42 on lipopolysaccharide (LPS)-stimulated inflammatory mediators in primary monocytes. Graphs show the effects of three different batches of viable *L. plantarum* GOS42 at different concentrations on interleukins (IL) -1β (a) and -6 (b), and prostaglandin E_2_ (PGE_2_) (c); results are from three independent experiments, each performed with biological triplicates and technical duplicates (*n* = 6 per experiment). All data are expressed as means ± standard deviation. The colony forming units per ml corresponding to each concentration for each batch are shown in Supplementary Table 4.

Viable and attenuated *L. paracasei* LPc-G110 and *L. plantarum* GOS42 induced dose-dependent reductions of LPS-stimulated release of the majority of the inflammatory mediators tested in primary monocytes (Figures 4 and 5). For both strains, the largest dose-dependent reductions were seen for IL-6, PGE_2_ and 8-isoprostane. Although substantial reductions in TNF-α were observed when either strain (viable or attenuated) was applied at 5% v/v, lower concentrations induced increases in TNF-α release. In the same experiment, these effects were consistent across different batches of viable and attenuated probiotics for both strains. The effects of different batches of viable *L. paracasei* LPc-G110 and *L. plantarum* GOS42 on IL-1β, for which the anti-inflammatory effects were minimal at concentrations up to 2.5% v/v, and on IL-6 and PGE_2_, for which they were substantial, are shown in Figures 6 and 7. The effects of both strains in viable and attenuated forms on all inflammatory mediators are shown in Supplementary Figures 1–4.

**Figure 8.**
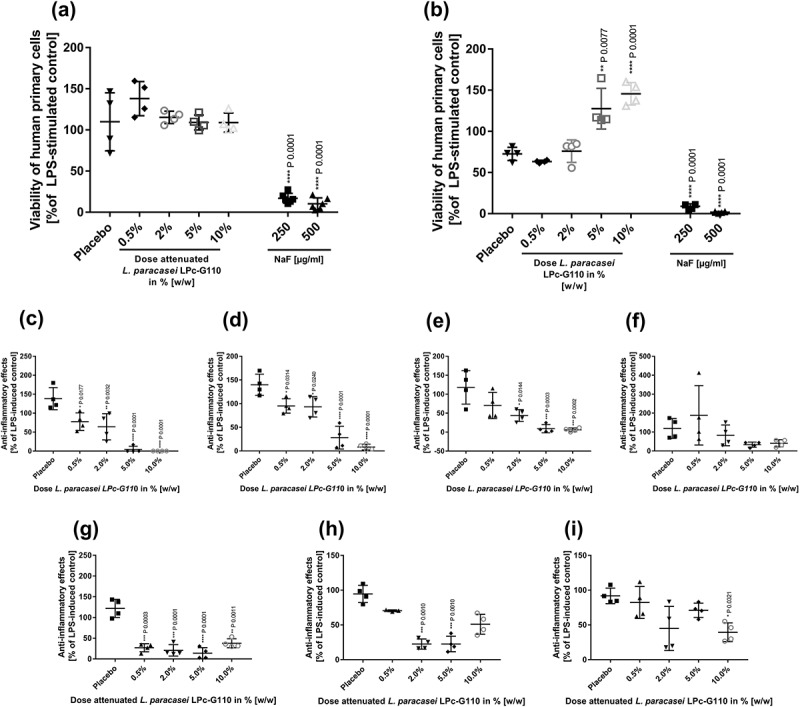
Anti-inflammatory effects of *Lactobacillus paracasei* LPc-G110 formulated in toothpaste. Viability of primary monocytes when exposed to different concentrations of viable (a) or attenuated (b) *L. paracasei* LPc-G110 toothpaste, or placebo toothpaste. Effects of viable *L. paracasei* LPc-G110 toothpaste on interleukins (IL) -6 (c) and -8 (d), PGE_2_ (e) and 8-isoprostane (f). Effects of attenuated *L. paracasei* LPc-G110 toothpaste on IL-6 (g), PGE_2_ (h) and 8-isoprostane (i). Results are from a single experiment with two biological and two technical replicates. *P* values are from matched one-way analysis of variance followed by Dunnett’s multiple comparisons test – compared to placebo control. The colony forming units per g corresponding to each concentration of each probiotic toothpaste are shown in Supplementary Table 5.

*L. paracasei* LPc-G110 was further evaluated for its functional consistency when formulated into toothpaste. The viability of *L. paracasei* LPc-G110 in the toothpaste after 2 months’ storage at 2–8°C was confirmed (Supplementary Table 5), and attenuated *L. paracasei* LPc-G110 toothpaste was confirmed to be free of viable probiotic bacteria. The cytotoxic effects of toothpaste, with or without probiotic, on primary monocytes were negligible up to 100 mg of toothpaste, as determined by the Alamar Blue-based cytotoxicity assay. Of note, a statistically significant increase in the viability of monocytes was seen in this assay for viable *L. paracasei* LPc-G110 toothpaste at concentrations of 5% and 10% w/w as Alamar Blue is not specific to human cells and the bacteria therefore contributed to the reaction (Figure 8(a)). Viable *L. paracasei* LPc-G110 toothpaste (100 mg) induced statistically significant dose-dependent reductions in the LPS-stimulated release of three (IL-6, IL-8 and PGE_2_) of the six inflammatory mediators, compared to the placebo control (100 mg), when administered at concentrations ranging from 0.5% to 10% (Figure 8(c, d, e)); a reduction in 8-isoprostane was also indicated but did not reach statistical significance (Figure 8(f)). Attenuated *L. paracasei* LPc-G110 toothpaste (100 mg) induced statistically significant reductions in LPS-stimulated IL-6, PGE_2_ and 8-isoprostane (Figure 8(g, h, i)).

## Discussion

This study has shown that specific strains of probiotic bacteria induce a dose-dependent downregulation of inflammatory mediators released by *ex-vivo* human monocytes in response to bacterial LPS, with some anti-inflammatory effects also seen in IL-1β-stimulated gingival fibroblasts. Moreover, further evaluation of two lead strains (*L. paracasei* LPc-G110, isolated from Mongolian fermented food and *L. plantarum* GOS42, from the oral cavity of a Swedish female student) that showed potent anti-inflammatory effects in initial screening, demonstrated their effects to be highly reproducible across probiotic batches, largely independent of strain viability and maintained (*L. paracasei* LPc-G110 only) when formulated into toothpaste. To the best of the authors’ knowledge, this is the first report of a dose-ranging study of probiotics for oral care.

In many cases, clinical investigations of probiotics for oral health are performed without any preclinical evidence of their beneficial potential in the oral cavity and the rationale for their use is based on their mechanisms of action in the gut [18]. The use of a screening strategy for probiotic strains, such as the one employed here, enables the rational selection of candidates with optimal activity for evaluation in clinical trials, wherein their therapeutic effects can be determined and safety assessed. Two lead strains have so far been selected for clinical testing among those evaluated in this study, but additional strains with similar properties may be selected after further evaluation. In addition to screening for anti-inflammatory activity, the probiotic strains evaluated in this study are being screened in parallel for antagonism to, and coaggregation with, disease-associated oral bacteria, and for their effects on the composition of oral biofilms in an *in vitro* model, with positive preliminary results reported [19,20]. As the oral microbiota is known to play an important role in health, the administration of probiotics with these potentially beneficial properties may be a viable approach to maintain or restore a healthy balance in the microbiota rather than indiscriminate eradication through the use of antimicrobials [9,21]. The probiotic screening strategy described here is versatile, so it could also be adapted and extended to other indications.

The results of the present study support previous studies showing that strains of commensal lactobacilli can suppress the release of inflammatory mediators from immune cells, and that these effects are strain specific [22–24]. Indeed, the strain specificity of the effects is highlighted by the fact that the majority of strains in the initial screen of this study did not show any anti-inflammatory activity, while the lead strains *L. paracasei* LPc-G110 and *L. plantarum* GOS42 inhibited the release of all six inflammatory mediators tested from monocytes. The mechanism by which these strains inhibited the LPS-stimulated release of inflammatory mediators from the host cells has not been elucidated and warrants investigation in future studies. However, previous research has shown that probiotic strains used in the gut can interact with toll-like receptors on host cells to downregulate the expression of nuclear factor-kappa-B and proinflammatory cytokines [25].

In the present study, LPS was used to stimulate an inflammatory response in monocytes in the model of gingival inflammation as it is a potent inflammatory agent, and LPS levels in GCF have been positively correlated with clinical and histological signs of gingivitis [26]. Moreover, LPS is a principal component of the outer membrane of Gram-negative bacteria, which increase in proportion in plaque during the development of gingivitis [1,2]. Monocytes and gingival fibroblasts were chosen as host cells in the model because both cell types play an important role in maintaining gingival tissue homeostasis and can secrete a variety of inflammatory mediators in response to different stimuli [5]. Since probiotics may have differing immunomodulatory effects on different host cell types, it is relevant to evaluate more than one type of host cell. A positive control, in the form of a known anti-inflammatory agent (e.g. a small molecule inhibitor), was not included in the model and this is a limitation of the study. However, comparison of such an agent with complex living organisms may be of questionable relevance.

The maintenance of anti-inflammatory effects in some strains, including *L. paracasei* LPc-G110 and *L. plantarum* GOS42, following their heat inactivation suggests that attenuated rather than viable probiotic bacteria could be used in oral care products. This finding is in line with the fact that toll-like receptors on immune and epithelial cells can recognize specific bacterial components from dead cells [27]. Despite viability being a requirement of a probiotic per the World Health Organization definition, use of attenuated probiotics could be advantageous due to compliance with regulatory demands, which include microbiological stability, as well as leading to a longer shelf-life of the final product.

Further evaluation of the two lead strains showed their anti-inflammatory effects, in most cases, to be dose dependent and consistent across different probiotic batches, despite the expected batch-to-batch fermentation variability in the Good Manufacturing Practice (GMP)-like production process used. As with the screening experiment, these effects were generally similar for both viable and attenuated forms of each strain. The anti-inflammatory effects of *L. paracasei* LPc-G110 and *L. plantarum* GOS42 were, therefore, highly reproducible. In addition, the effects of strain *L. paracasei* LPc-G110 were maintained when it was formulated into toothpaste, as evidenced by the dose-dependent reduction in multiple inflammatory mediators compared to the placebo control. The use of the excipient toothpaste as the placebo control for comparison ensured that the observed effects of the probiotic were not a result of the excipient itself. Indeed, at the mass used, the excipient had negligible effects on the viability of the monocytes and release of the inflammatory mediators tested. These results provide a preclinical proof of principle for the formulation of functionally active probiotics in toothpaste.

In conclusion, strains *L. paracasei* LPc-G110 and *L. plantarum* GOS42 showed potent anti-inflammatory effects, which were largely independent of their viability and, in the case of *L. paracasei*, were maintained when formulated into toothpaste. These strains therefore have potential for use as probiotics in oral care products to reduce gingival inflammation. A proof-of-principle clinical study to determine whether these strains are beneficial for the maintenance of gingival health versus placebo in healthy volunteers refraining from normal oral hygiene is planned.
