# Supplementary material for: Ex vivo anti-inflammatory effects of probiotics for periodontal health
Source: J Oral Microbiol. 2018 Jul 25;10(1):1502027. doi: 10.1080/20002297.2018.1502027 (PMC6060379; doi:10.1080/20002297.2018.1502027)
Supplement: Supplemental Material [file ZJOM_A_1502027_SM8178.docx]

Supplementary Table 1. Viable counts of *Lactobacillus paracasei* LPc-G110 and *Lactobacillus plantarum* GOS42 in Figure 1 – screening in monocytes

| **Strain** | ***L. paracasei* LPc-G110 [CFU/ml]** | ***L. plantarum* GOS42 [CFU/ml]** |
| --- | --- | --- |
| **Batch** | 20130504 | td16/5-2013 |
| Stock | 870 x 10^5^ | 131 x 10^8^ |
| 5.0% [v/v] | 43.5 x 10^5^ | 6.55 x 10^8^ |
| 1.0% [v/v] | 8.7 x 10^5^ | 1.31 x 10^8^ |
| 0.5% [v/v] | 4.35 x 10^5^ | 0.655 x 10^8^ |
| 0.1% [v/v] | 0.87 x 10^5^ | 0.131 x 10^8^ |

CFU = colony forming units; v = volume

Supplementary Table 2. Viable counts of *Lactobacillus paracasei* LPc-G110 and *Lactobacillus plantarum* GOS42 in Figures 2 and 3 – screening in gingival fibroblasts

| **Strain** | ***L. paracasei* LPc-G110 [CFU/ml]** | ***L. plantarum* GOS42 [CFU/ml]** |
| --- | --- | --- |
| **Batch** | 20130504 | td16/5-2013 |
| Stock | 1200 x 10^5^ | 360 x 10^6^ |
| 5.0% [v/v] | 60 x 10^5^ | 18 x 10^6^ |
| 2.5% [v/v] | 30 x 10^5^ | 9 x 10^6^ |
| 1.0% [v/v] | 12 x 10^5^ | 3.6 x 10^6^ |
| 0.5% [v/v] | 6 x 10^5^ | 1.8 x 10^6^ |
| 0.1% [v/v] | 1.2 x 10^5^ | 0.36 x 10^6^ |

CFU = colony forming units; v = volume

Supplementary Table 3. Viable counts of *Lactobacillus paracasei* LPc-G110 in Figures 4 and 6, and Supplementary Figures 1 and 3 – dose-finding and batch-to-batch variation in primary monocytes

| **Strain** | ***L. paracasei* LPc-G110 [CFU/ml]** | | | |
| --- | --- | --- | --- | --- |
| **Batch** | 1 (20130504) | 2 (2015-09-18) | 3 (2015-09-20) | 4 (2015-09-24) |
| Stock | 232 x 10^6^ | 295 x 10^6^ | 430 x 10^6^ | 290 x 10^6^ |
| 5.00% [v/v] | 11.6 x 10^6^ | 14.75 x 10^6^ | 21.5 x 10^6^ | 14.5 x 10^6^ |
| 2.50% [v/v] | 5.8 x 10^6^ | 7.375 x 10^6^ | 10.75 x 10^6^ | 7.25 x 10^6^ |
| 1.00% [v/v] | 2.32 x 10^6^ | 2.95 x 10^6^ | 4.3 x 10^6^ | 2.9 x 10^6^ |
| 0.75% [v/v] | 1.74 x 10^6^ | 2.2125 x 10^6^ | 3.225 x 10^6^ | 2.175 x 10^6^ |
| 0.50% [v/v] | 1.16 x 10^6^ | 1.475 x 10^6^ | 2.15 x 10^6^ | 1.45 x 10^6^ |
| 0.25% [v/v] | 0.58 x 10^6^ | 0.7375 x 10^6^ | 1.075 x 10^6^ | 0.725 x 10^6^ |
| 0.10% [v/v] | 0.232 x 10^6^ | 0.295 x 10^6^ | 0.43 x 10^6^ | 0.29 x 10^6^ |
| 0.05% [v/v] | 0.116 x 10^6^ | 0.1475 x 10^6^ | 0.215 x 10^6^ | 0.145 x 10^6^ |

CFU = colony forming units; v = volume

Supplementary Table 4. Viable counts of *Lactobacillus plantarum* GOS42 in Figures 5 and 7, and Supplementary Figures 2 and 4 – dose-finding and batch-to-batch variation in primary monocytes

| **Strain** | ***L. plantarum* GOS42 [CFU/ml]** | | |
| --- | --- | --- | --- |
| **Batch** | 1 (td16/5-2013) | 2 (C117896A) | 3 (C101231S) |
| Stock | 550 x 10^6^ | 232 x 10^6^ | 1150 x 10^6^ |
| 5.00% [v/v] | 27.5 x 10^6^ | 11.6 x 10^6^ | 57.5 x 10^6^ |
| 2.50% [v/v] | 13.75 x 10^6^ | 5.8 x 10^6^ | 28.75 x 10^6^ |
| 1.00% [v/v] | 5.5 x 10^6^ | 2.32 x 10^6^ | 11.5 x 10^6^ |
| 0.75% [v/v] | 4.125 x 10^6^ | 1.74 x 10^6^ | 8.625 x 10^6^ |
| 0.50% [v/v] | 2.75 x 10^6^ | 1.16 x 10^6^ | 5.75 x 10^6^ |
| 0.25% [v/v] | 1.375 x 10^6^ | 0.58 x 10^6^ | 2.875 x 10^6^ |
| 0.10% [v/v] | 0.55 x 10^6^ | 0.232 x 10^6^ | 1.15 x 10^6^ |
| 0.05% [v/v] | 0.275 x 10^6^ | 0.116 x 10^6^ | 0.575 x 10^6^ |

CFU = colony forming units; v = volume

Supplementary Table 5. Viable counts of lyophilized viable *Lactobacillus paracasei* LPc-G110 toothpaste

| **Component** | **Probiotic content** | | | | |
| --- | --- | --- | --- | --- | --- |
|  | **Placebo** | **0.50%** | **2.00%** | **5.00%** | **10.00%** |
| Calculated CFU/g |  | 7.2E+08 CFU/g | 2.9E+09 CFU/g | 7.2E+09 CFU/g | 1.4E+10 CFU/g |
| Mean CFU/g after 2 months storage^a^ |  | 1.2E+05 CFU/g | 2.2E+06 CFU/g | 5.2E+07 CFU/g | 3.6E+08 CFU/g |
| ^a^Viable counts were performed in triplicate  CFU: colony forming units | | | | | |


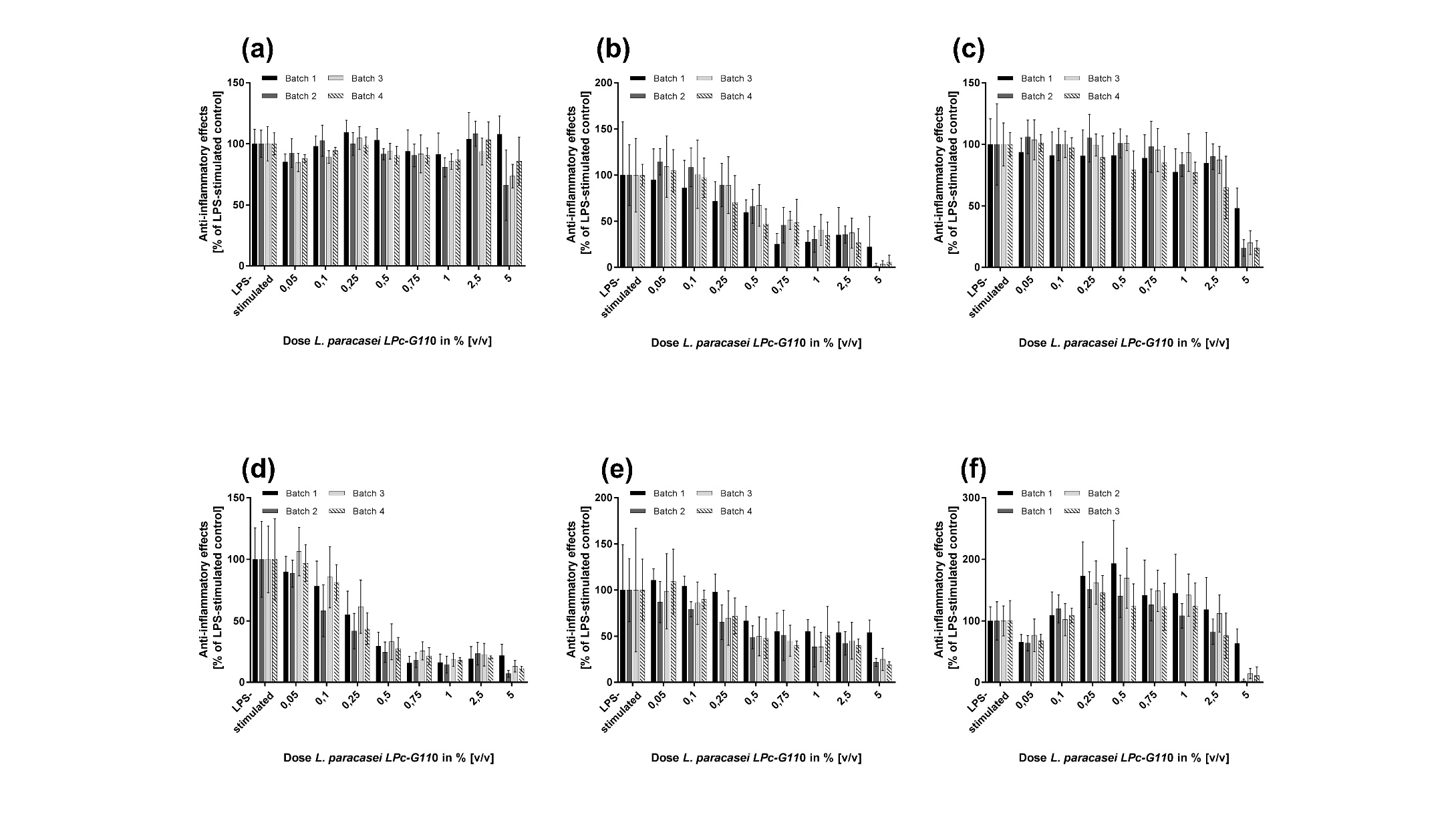


**Supplementary Figure 1.** **Batch-to-batch variation of effects of viable *Lactobacillus paracasei* LPc-G110 on lipopolysaccharide (LPS)-stimulated inflammatory mediators in primary monocytes.** Graphs show the effects of four different batches of viable *L. paracasei* LPc-G110 at different concentrations on interleukins (IL) -1β **(a)**, -6 **(b)** and -8 **(c)**, and prostaglandin E_2_ (PGE_2_) **(d)**, 8-isoprostane **(e)** and tumor necrosis factor (TNF)-α **(f)**; results are from four independent experiments, each performed with biological triplicates and technical duplicates (n=6 per experiment). All data are expressed as means ± standard deviation. The colony forming units per ml corresponding to each concentration for each batch are shown in Supplementary Table 3.

**
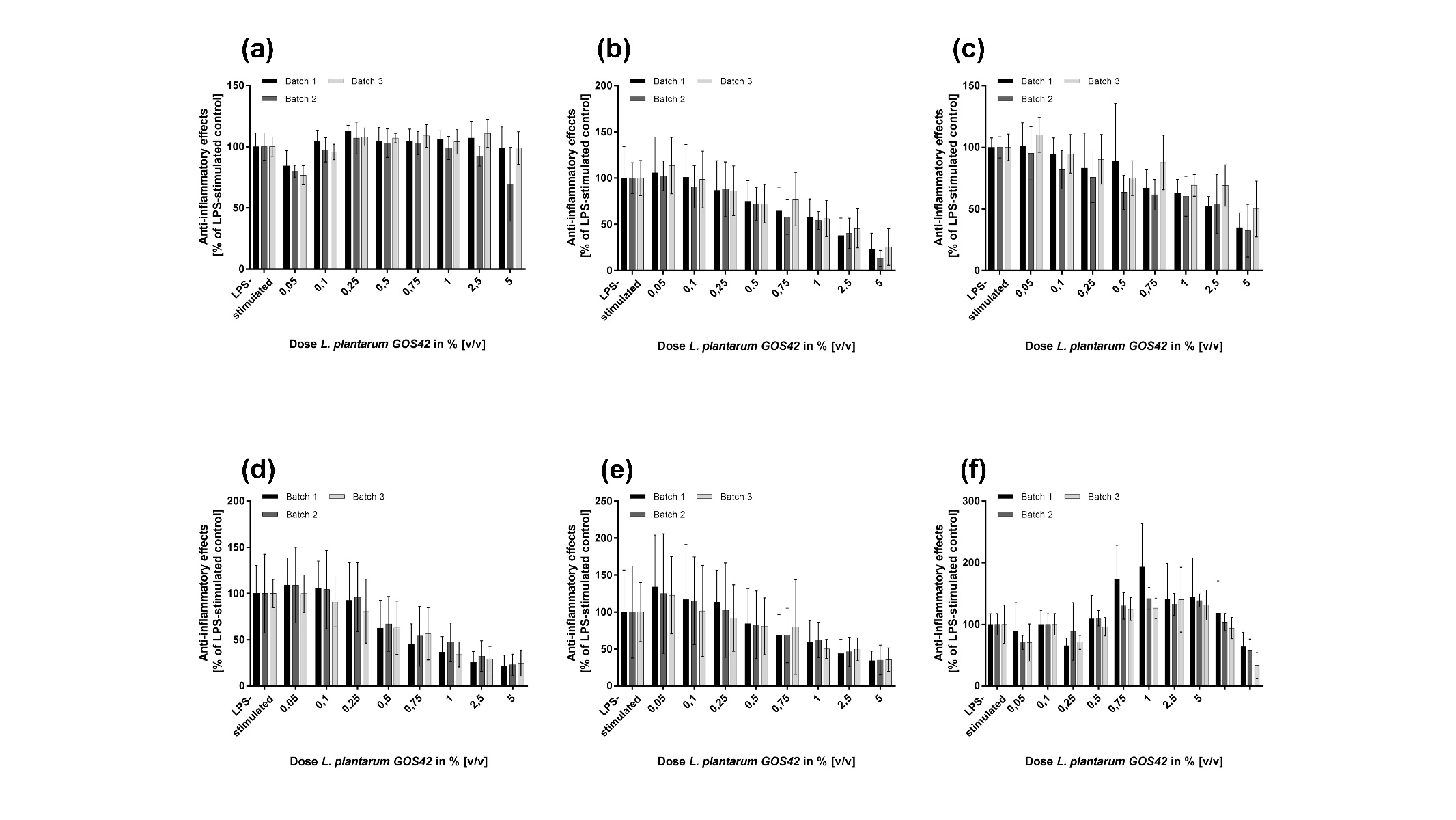
**

**Supplementary Figure 2. Batch-to-batch variation of effects of viable *Lactobacillus plantarum* GOS42 on lipopolysaccharide (LPS)-stimulated inflammatory mediators in primary monocytes.** Graphs show the effects of three different batches of viable *L. plantarum* GOS42 at different concentrations on interleukins (IL) -1β **(a)**, -6 **(b)** and -8 **(c)**, prostaglandin E_2_ (PGE_2_) **(d)**, 8‑isoprostane **(e)** and tumor necrosis factor (TNF)-α **(f)**; results are from three independent experiments, each performed with biological triplicates and technical duplicates (n=6 per experiment). All data are expressed as means ± standard deviation. The colony forming units per ml corresponding to each concentration for each batch are shown in Supplementary Table 4.


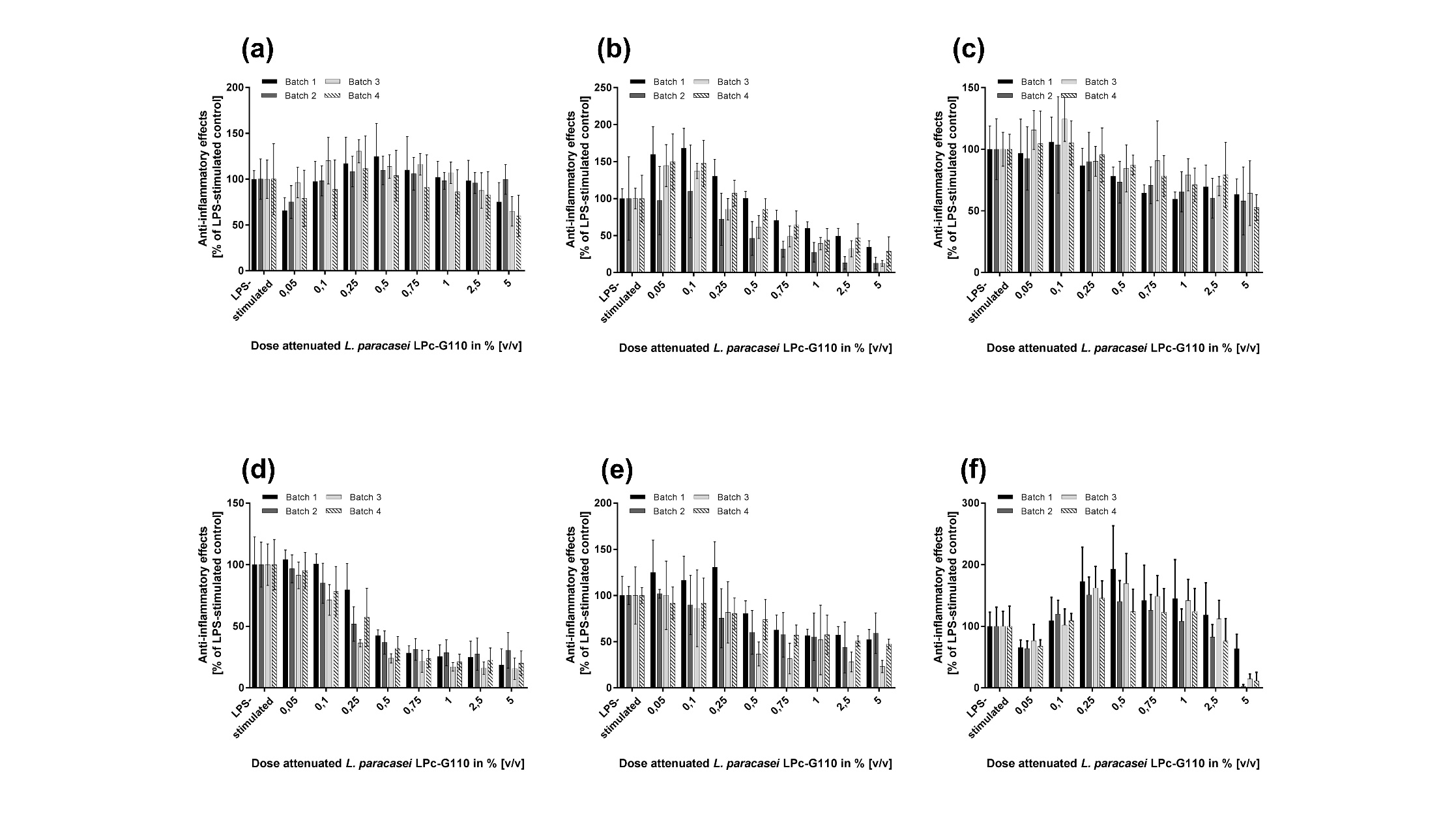


**Supplementary Figure 3. Batch-to-batch variation of effects of attenuated *Lactobacillus paracasei* LPc-G110 on lipopolysaccharide (LPS)-stimulated inflammatory mediators in primary monocytes.** Graphs show the effects of four different batches of attenuated *L. paracasei* LPc-G110 at different concentrations on interleukins (IL) -1β **(a)**, -6 **(b)** and -8 **(c)**, prostaglandin E_2_ (PGE_2_) **(d)**, 8-isoprostane **(e)** and tumor necrosis factor (TNF)-α **(f)**; results are from four independent experiments, each performed with biological triplicates and technical duplicates (n=6 per experiment). All data are expressed as means ± standard deviation. The colony forming units per ml corresponding to each concentration for each batch are shown in Supplementary Table 3.


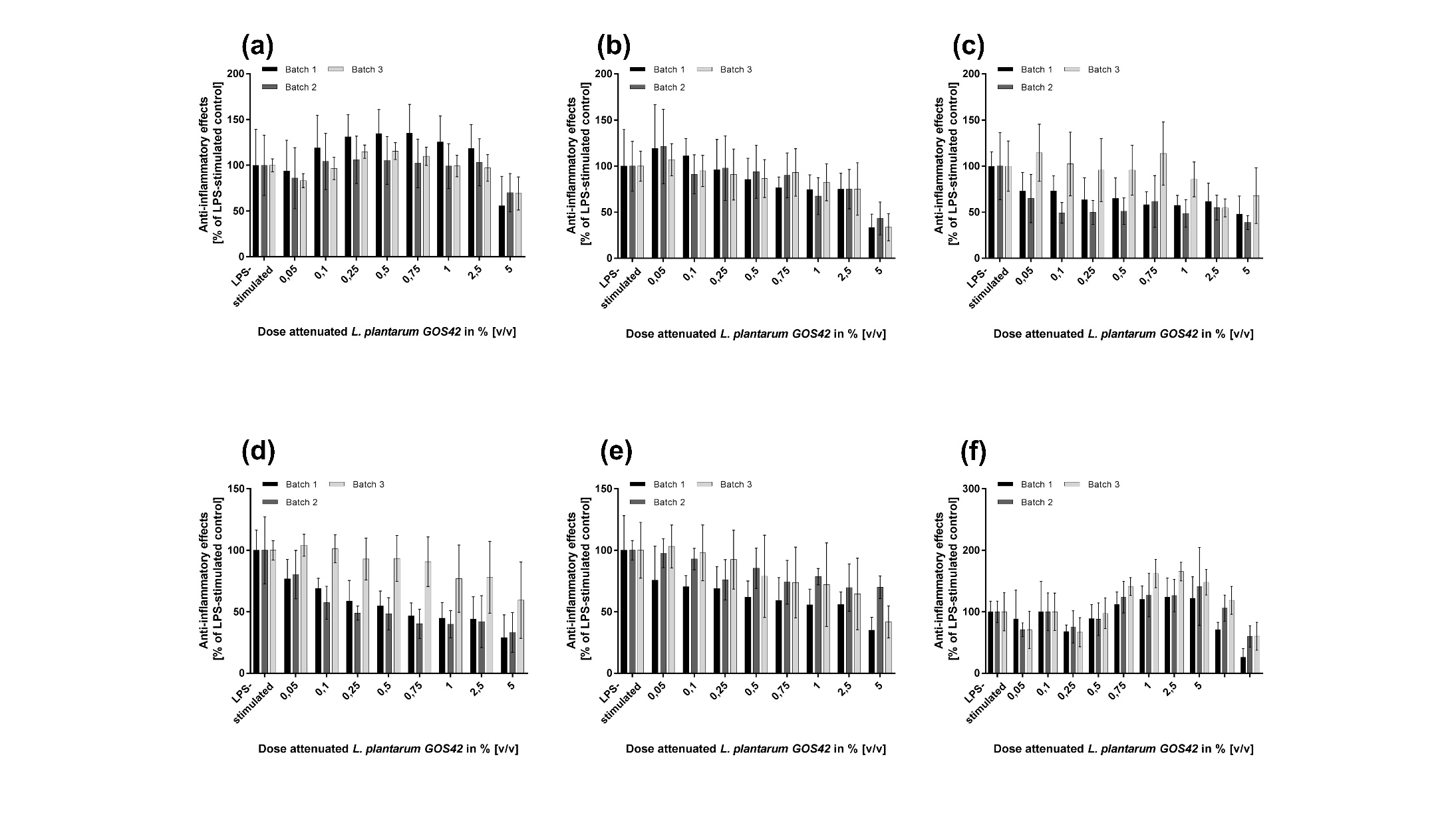


**Supplementary Figure 4. Batch-to-batch variation of effects of attenuated *Lactobacillus plantarum* GOS42 on lipopolysaccharide (LPS)-stimulated inflammatory mediators in primary monocytes.** Graphs show the effects of three different batches of attenuated *L. plantarum* GOS42 at different concentrations on interleukins (IL) -1β **(a)**, -6 **(b)** and -8 **(c)**, prostaglandin E_2_ (PGE_2_) **(d)**, 8‑isoprostane **(e)** and tumor necrosis factor (TNF)-α **(f)**; results are from three independent experiments, each performed with biological triplicates and technical duplicates (n=6 per experiment). All data are expressed as means ± standard deviation. The colony forming units per ml corresponding to each concentration for each batch are shown in Supplementary Table 4.
